# Incomplete bioinformatic filtering and inadequate age and growth analysis lead to an incorrect inference of harvested‐induced changes

**DOI:** 10.1111/eva.13122

**Published:** 2020-09-12

**Authors:** Wesley A. Larson, Daniel A. Isermann, Zachary S. Feiner

**Affiliations:** ^1^ National Oceanographic and Atmospheric Administration National Marine Fisheries Service Alaska Fisheries Science Center Auke Bay Laboratories Juneau AK USA; ^2^ U.S. Geological Survey Wisconsin Cooperative Fishery Research Unit College of Natural Resources University of Wisconsin‐Stevens Point Stevens Point WI USA; ^3^ Wisconsin Department of Natural Resources Office of Applied Science Science Operations Center Madison WI USA

**Keywords:** age and growth analysis, bioinformatic filtering, fisheries harvest, population genomics, RADseq, walleye

## Abstract

Understanding the evolutionary impacts of harvest on fish populations is important for informing fisheries management and conservation and has become a growing research topic over the last decade. However, the dynamics of fish populations are highly complex, and phenotypes can be influenced by many biotic and abiotic factors. Therefore, it is vital to collect robust data and explore multiple alternative hypotheses before concluding that fish populations are influenced by harvest. In their recently published manuscript, Bowles et al, Evolutionary Applications, 13(6):1128 conducted age/growth and genomic analysis of walleye (*Sander vitreus*) populations sampled 13–15 years (1–2.5 generations) apart and hypothesized that observed phenotypic and genomic changes in this time period were likely due to harvest. Specifically, Bowles et al. (2020) documented differential declines in size‐at‐age in three exploited walleye populations compared to a separate, but presumably less‐exploited, reference population. Additionally, they documented population genetic differentiation in one population pair, homogenization in another, and outlier loci putatively under selection across time points. Based on their phenotypic and genetic results, they hypothesized that selective harvest had led to fisheries‐induced evolution (referred to as nascent changes) in the exploited populations in as little as 1–2.5 generations. We re‐analyzed their data and found that (a) sizes declined across both exploited and reference populations during the time period studied and (b) observed genomic differentiation in their study was the result of inadequate data filtering, including retaining individuals with high amounts of missing data and retaining potentially undersplit and oversplit loci that created false signals of differentiation between time points. This re‐analysis did not provide evidence for phenotypic or genetic changes attributable to harvest in any of the study populations, contrasting the hypotheses presented by Bowles et al. (2020). Our comment highlights the potential pitfalls associated with conducting age/growth analyses with low sample sizes and inadequately filtering genomic datasets.

## 1 INTRODUCTION

Understanding whether harvest impacts the long‐term viability of fish populations is vital for informing conservation and fisheries management (Dunlop, Eikeset, & Stenseth, 2015). Fortunately, this important topic is becoming more well‐studied as fisheries managers are beginning to understand that harvest can threaten the long‐term viability of populations even if population abundances are not decreasing (Hutchings & Kuparinen, 2020). Bowles, Marin, Mogensen, MacLeod, and Fraser (2020) add to the growing body of literature on this topic by investigating potential phenotypic and genetic changes resulting from harvest in populations of walleye (*Sander vitreus)* from Mistassini Lake, a large lake in Quebec, Canada. The paper documents phenotypic and genomic changes in these walleye populations over 1–2.5 generations that the authors hypothesize are likely due to harvest, a striking result given that most other studies do not find impacts of harvest on this short of a timescale. However, we believe that these conclusions are supported by inadequate age/growth data and genomic evidence that disappears when data are filtered correctly. We outline some specific issues with the age/growth and genomic data below and argue that if the data were analyzed correctly, the conclusions presented by the authors would not have been reached.

## 2 BODY SIZE ANALYSIS

We agree that the Indigenous Knowledge (IK) obtained by the authors supports the assertion that harvest has affected size structure of walleye stocks examined in the study. However, whether those reductions can be directly attributed to selective removal of specific genotypes or phenotypes from a wild population, and that harvest differentially affected southern and northern walleye populations, requires robust data and sampling that occurs at least somewhat consistently over time (Dieckmann & Heino, 2007; Sinclair, Swain, & Hanson, 2002). Size distributions and length‐at‐age in individual walleye populations vary extensively on a temporal scale (Hansen & Nate, 2014; Isermann, 2007; Wuellner, Willis, Blackwell, & Lott, 2011) and can vary among populations or stocks at relatively small spatial scales (Pedersen et al., 2017; Sass, Hewett, Beard, Fayram, & Kitchell, 2004; Wang et al., 2012). Differences in recruitment, density, and environmental conditions are just a few of the many factors that have been shown to influence walleye size distributions or length‐at‐age (Lester, Shuter, Kushneriuk, & Marshall, 2000; Pedersen et al., 2017; Quist, Guy, Schultz, & Stephen, 2003; Sass & Kitchell, 2005). Changes in harvest may or may not affect size structure in walleye populations (Haglund, Isermann, & Sass, 2016; Hansen & Nate, 2014; Sass & Shaw, 2018, 2020; Sullivan, 2003); at a fundamental level, the effects of harvest on fish populations will vary in relation to abundance and exploitation rate (i.e., percentage of population harvested on annual basis).

In an experimental sense, Bowles et al. (2020) must show that growth trends differed between the southern systems and northern reference system and that the only factor that changed between the two was walleye harvest. This can be done in whole lake experiments with explicit control of harvest (e.g., Haglund et al., 2016; Sass & Shaw, 2018), but is much more difficult in wild populations. Mistassini Lake is a large system, and the presumably low exploitation reference tributary (Takwa River) is located more than 150 km from the high exploitation tributaries in the south; this latitudinal separation alone is sufficiently large to suggest that the reference population might encounter different environmental conditions and exhibit different population dynamics than walleye farther south (as discussed in Dupont, Bourret, & Bernatchez, 2007). Indeed, Bowles et al. (2020) report that walleye spawning in southern tributaries “stay close” to these locations after spawning, represent the majority of fish harvested during the mixed fishery, and exhibit different growth patterns than northern fish (Dupont et al., 2007). Therefore, it is plausible that these populations were experiencing different environmental conditions throughout the study period that could influence life history trait expression.

**FIGURE 1:**
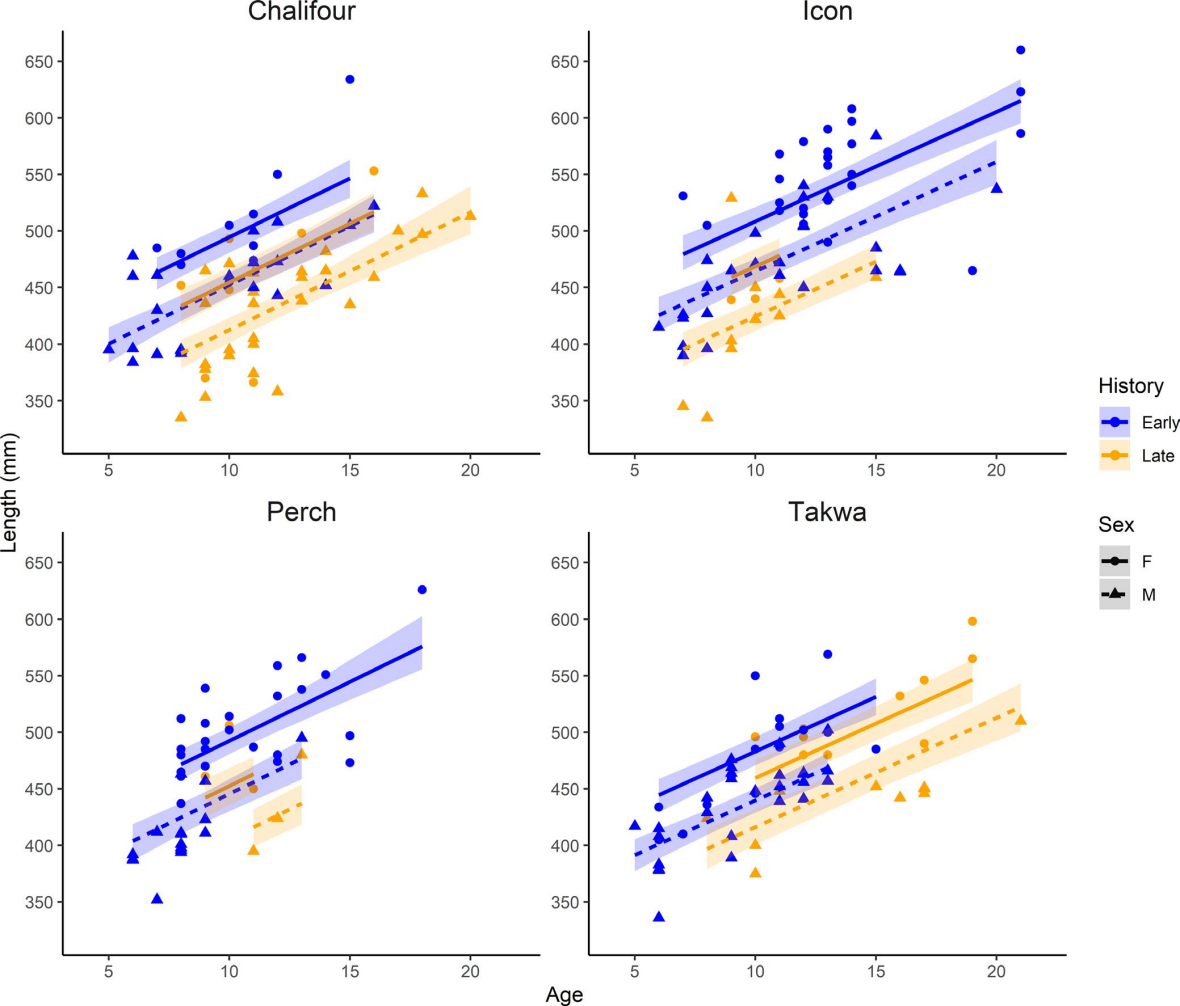
Length‐at‐age observations (points) and predicted model fits (lines with shaded 95% credible intervals) for walleye captured from the four tributaries documented in Bowles et al. (2020). Observations are color‐coded by time period (historical, 2002 = blue; contemporary, 2015/2017 = orange) and sex (female = circles and solid lines, males = triangles and dashed lines). Length‐at‐age declined from the historical to contemporary period in all four populations

Because of these complexities, the reliance of Bowles et al. (2020) on two observations of walleye length‐at‐age 12 years apart (2003 to 2015) provides little context for the variability or co‐variability of growth among study populations. The authors provide no quantifiable information regarding abundance and exploitation rates of the walleye populations in question, or the size selectivity of fishers, making it impossible to discern the effects of harvest. Moreover, examination of the otoliths (conducted by Isermann's research group at University of Wisconsin‐Stevens Point) revealed that the age structure of walleye from these tributaries was dominated by fish older than age 10 with a relatively high proportion of fish age 15 or older. In a species with an approximate maximum age of 20 years (including these populations, Figure 1; Venturelli, Lester, Marshall, & Shuter, 2010), this suggests that overall mortality for the study populations was relatively low and growth, maturation, and susceptibility to harvest was delayed (Lavigne, Lucotte, & Paquet, 2010). The lack of demographic and ecological context for these populations at a sufficiently fine temporal scale leads to a number of unanswered questions that are essential to addressing the authors’ hypotheses. Did age composition change over time? How extensive was exploitation in each study population? Does exploitation occur at levels that might be expected to cause selective pressures, and is size selectivity sufficiently intense? What if some stocks were historically more abundant than others? What if exploitation rates vary significantly among populations and among years within a population? Did size distributions and length‐at‐age of walleye from southern tributaries fluctuate over this period or were there detectable trends in these metrics that would at least suggest the possibility that harvest might be exerting an effect?

To expand on one of these questions as an example, declines in body size over time could be rooted in harvest, but could also reflect loss of older, larger fish from strong year classes that may have been produced more than a decade before samples were collected based on the age distribution of the walleye examined. In their reporting of harvest, Bowles et al. (2020) note that harvest was 54% higher in 2003 but varied little in other years from 1997, 2011, and 2015 (Table S2), despite increases in the local indigenous population. The production of a large year class in the years preceding 2003 could have led to increased harvest observed in 2003, reduced growth through density‐dependent mechanisms of subsequent cohorts, and a perceived reduction in old/large fish in the study's contemporary sampling as those cohorts would have comprised the older fish sampled for ages in 2015–2017. Therefore, based on the ages of fish collected by the authors, walleye abundance and size structure in the contemporary period were undoubtedly affected by recruitment events that occurred during the historic period. The authors never mention this important fact. Additionally, correlations among growth, mortality, and maturation complicate the interpretation of growth differences because the fish included in the study were all sampled while spawning. Walleye maturation is highly plastic (Feiner & Höök, 2015), and increased growth, condition, or energy reserves can increase the probability that small walleyes will mature and spawn (Feiner, Shaw, & Sass, 2019; Henderson & Morgan, 2002). If fishing or other factors (e.g., temperature, food availability) affected growth or condition of walleye in the lake, individuals experiencing more favorable environmental conditions may become mature at smaller sizes for a given age, leading to the phenomenon of catching smaller fish on the spawning grounds as observed by Bowles et al. (2020) in the absence of any selective or genetic impacts of fishing itself. The authors did not account for any of these other factors in their analyses, and yet they suggest the differences they observed in their size and growth metrics may be the result of harvest‐induced selection occurring within 1–2.5 generations. At best, harvest‐induced evolution would be one of many factors that may explain the trends the authors observed, and there is no way to reliably identify the mechanisms driving size trends given the authors’ study design.

## 3 LENGTH‐AT‐AGE ANALYSIS

Because of the issues with the body size analysis we outlined above, evidence for phenotypic effects due to harvest in this study hinges on the length‐at‐age analysis. Our issues with the length‐at‐age analysis are twofold: First, we argue that the data are insufficient to examine patterns in growth, and second, we argue their length‐at‐age model does not show differential growth patterns between southern and northern walleye populations. Length‐at‐age analysis typically involves a stratified‐random sampling approach where calcified structures are removed for age estimation from a specific number of fish of each sex within defined length categories (i.e., structures removed from 10 fish of each sex per 10‐mm length group) and ages from these fish are extrapolated back to the entire sample (Coggins, Gwinn, & Allen, 2013). However, in Bowles et al. (2020), there appears to be no specific sampling target defined by total number of fish or percentage of overall samples, and sample sizes are generally small. The total number of walleye selected for age estimation in a year from a specific tributary was less than 25 in five of the ten annual samples that were used in analyses and was greater than or equal to 50 in only two instances, indicating that tributary‐specific sample sizes for length‐at‐age were likely less than five for many ages. In the most extreme case, the contemporary age sample for the Perch River is reported as six fish total across all ages. For female walleye, total sample sizes used to characterize length‐at‐age over the three‐year “contemporary” sampling period never exceeded 11 fish for any tributary. Attempting to characterize female length‐at‐age for a population using 11 fish or fewer in a single year is not reasonable, let alone over a three‐year period. Therefore, sample sizes were insufficient for a legitimate analysis of female walleye body size or length‐at‐age.

The authors attempted to account for these issues, in addition to potential biases due to sampling gear and aging structures, by using a Bayesian hierarchical modeling approach. However, Bayesian models, while better able to handle difficult data distributions or hierarchical designs, are not a cure for flawed or insufficient sampling—the data are the data (Lecoutre, 1999; O'Hagan, 2006). For instance, error terms for sampling gear or aging structure are not included and the model appears to assume that the effects of age and sex are independent, which is likely violated (Feiner et al., 2017). Most importantly, the model provides no direct comparison of length‐at‐age among populations, even though this is the phenotypic shift that represents the crux of the paper's argument. Rather, the length‐at‐age model assesses whether variation in length of walleye (not length‐at‐age) can be explained by age, sampling period (historic versus. contemporary), location, or sex, essentially a duplication of their previous analyses regarding length of fish but using only the fish that were selected for age estimation. Here, assuming all other ecological factors remained constant, the specific questions of interest are 1) are walleye from southern tributaries now smaller at a given age (e.g., age 11) than they were in the historic period?, and 2) was this trend also apparent for fish from the reference tributary? At a minimum, an age*location*history interaction term would be needed to test whether the effect of age (i.e., growth) differed between locations and histories. However, the results of the model are unclear. In the Results, the model is described as suggesting “fish were 29.4 mm larger contemporaneously than historically” and “fish in southern rivers were 13.7 mm smaller relative to fish in the north in contemporary relative to historical samples.” If the first statement is accurate, this refutes the hypothesis that harvest negatively influenced growth over time. Moreover, the 13.7 mm difference in temporal changes in size between locations was marginal, with a confidence interval that overlapped zero with 13% of the posterior less than zero. This suggests, as we do, that the phenotypic shifts in body size were likely caused by ecological mechanisms.

We re‐analyzed the length‐at‐age data in an attempt to duplicate Bowles et al.’s (2020) results and test for contrasts in the predicted size of age‐11 fish across populations and time periods to address the questions we posed above. Data were retrieved from the Dryad repository https://doi.org/10.5061/dryad.5tb2rbp1z and the file “Bowles_et_al‐Evol_App‐2020‐Age_infoR.xlsx.” We used a hierarchical Bayesian model parameterized following the one described in Bowles et al. (2020); however, our results did not match the reported results in the paper, we suspect because of some differences in model parameterization based on the relatively brief description of the model in the paper. In our model, we included random intercepts and slopes for the effect of age by river, random intercepts for the effect of sex by river, and fixed effects of the categorical variables for location (north or south) and history (historical or contemporary):

$$\begin{array}{c} \mu ∼\left(\beta_{0}+u_{0j}+\beta_{2j}\times \text{sex}\right)\;+\;\left(\beta_{1}+u_{1j}\right)\times \text{age}\;+\beta_{3i}+\beta_{4k}+\beta_{5i,k}\\ \text{Length}\;∼N(\mu ,\;\sigma ) \end{array}$$

where β_0_ is the grand mean, *u_0_* and *u_1_* are the random intercepts and slopes for age by river *j*, *β_2_* are the random effects of sex by river *j*, distributed around a mean effect $\beta_{2j}∼N\left(\mu_{\beta_{2}},\sigma_{\beta_{2}}\right)$, *β_3_* is the effect of location *k*, *β_4_* is the effect of history *k*, and *β_5_* is the interaction between location *i* and history *k*. We used a vague Cholesky prior for the random intercepts and slopes of age, half‐Cauchy priors for other parameter variances, and vague normal priors for parameter means. We calculated the predicted length of an age‐11 fish for each time period and location and compared the change between periods between north and south locations. We ran the model for 10,000 steps with a 5,000 warmup, thinning to every 10th step, and assured convergence using traceplots, effective sample size >1,000, and Gelman‐Rubin statistics approaching 1. The model was fit in Stan in R via package “rstan” (Carpenter et al., 2017; R & Development Core Team, 2019; Stan & Development Team, 2020). Model code is available in supplemental file 1.

Our model predicted that all populations, including the reference (“unfished”) population, were reduced in size‐at‐age from historical to contemporary periods (Figure 1; see Table S1 for all parameter estimates). Age‐11 fish in the north (i.e., Takwa) declined by approximately 24 mm (CI: 5.4–41.2 mm) in total length, while fish in the south (i.e., Chalifour, Perch, and Icon) declined in length by 40 mm (CI: 28.5–51.5 mm). The 95% credible intervals for this contrast in changes in length (South–North) overlapped zero (mean = 16 mm, CI: −4.2–36.1 mm) suggesting that differences in changes in size‐at‐age between regions may be marginal and reducing the strength of Bowles et al.’s argument that growth (measured by size‐at‐age) changed differentially among populations over time. Importantly, our re‐analysis of these data illustrated that size‐at‐age was potentially reduced in both the northern (low exploitation) and southern (high exploitation) areas of the lake, providing evidence that this trend may be the result of similar effects of harvest in both populations, and/or environmental factors.

## 4 Genomic analysis

The authors make three primary conclusions regarding the genetic characteristics of the walleye populations in their study: (a) genetic homogenization has occurred between two southern (i.e., heavily exploited) rivers over the study period (PER and ICO), (b) the genetic structure in at least one southern river (CHA) has increased and become significant, while no changes were observed in the northern (i.e., less exploited) river, and (c) there is evidence of selection between historic and contemporary samples in southern rivers. We re‐analyzed the data presented in Bowles et al. (2020) and found that all three of these conclusions are likely the result of incorrect bioinformatic processing and/or inadequate data filtering. Specifically, we found that the apparent genetic homogenization between populations was likely the result of high levels of missing data (>50% for all individuals) in the PER03 sample and that signatures of population differentiation and selection were largely driven by low *F*_IS_/high *F*_ST_ loci that are likely bioinformatic artifacts.

Understanding how different aspects of genomics datasets can influence downstream analyses is vital for conducting robust genomics research. This is especially true for datasets with low genetic structure, where small data inconsistencies such as differences in missing data or library effects can create patterns that appear to represent true population structure. Recently, O'Leary, Puritz, Willis, Hollenbeck, and Portnoy (2018) published an excellent overview of RAD data filtering, where they highlighted multiple potential issues with RAD datasets, how these issues might influence downstream analyses, and how to identify and mitigate these issues. Two particular issues raised in O'Leary et al. (2018), inconsistent sequencing of loci (i.e., missing data) and clustering errors (i.e., oversplitting or undersplitting loci), are likely the cause of the spurious results reported in Bowles et al. (2020).

**FIGURE 2:**
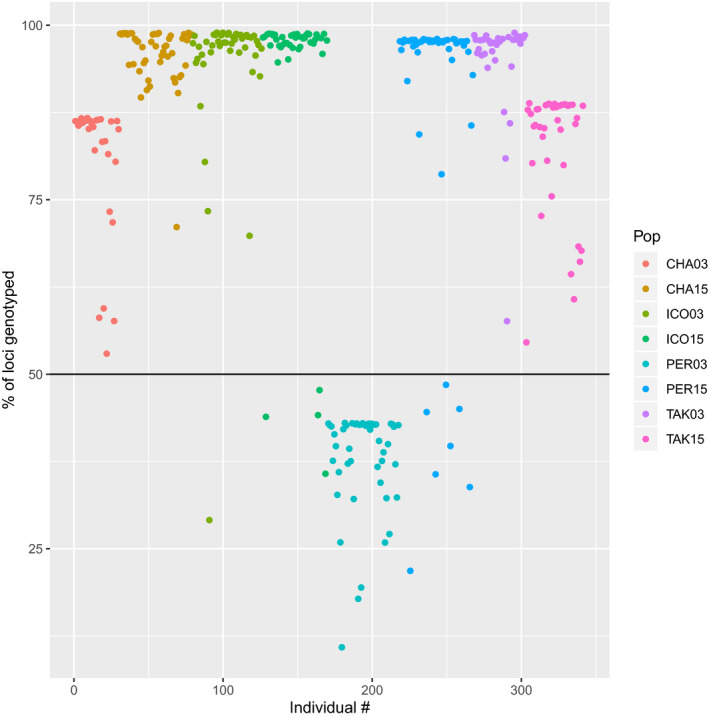
Percentage of missing data for all individuals in the dataset. Each individual is represented by a dot, and populations are color‐coded. See text for population abbreviations

One of the first steps for data filtering recommended by O'Leary et al. (2018) is a visualization of missingness across loci and individuals. We conducted this analysis for the Bowles dataset (Figure 2, Figure S1) using the Bowles_et_al‐EvolApp‐2020‐r12dWithOutOddBallSamps_includ‐outlier_loci.genepop file from the DRYAD repository for this paper https://doi.org/10.5061/dryad.5tb2rbp1z. This dataset was used as the starting point for all analyses in our comment, all R code used to analyze the data are in supplementary files, and population abbreviations used throughout the comment are as follows: Chalifour (CHA), Icon (ICO), Perch (PER), and Takwa (TAK) with year appended (i.e., PER03 are walleye from the 2003 samples of the Perch River). Unfortunately, the raw sequence data and sex information were not available in the supplementary materials, so we were unable to replicate all of the authors analyses (i.e., sex‐specific analysis, STACKs analysis). Genotyping rates were relatively consistent within populations but variable across populations (Figure 2). Most notably, all individuals in the PER03 population had >50% missing data, with some individuals displaying nearly 90% missing data, while the other populations sequenced relatively well (<20% missing data on average). The authors did not impose any missing data filter to remove individuals that were sequenced poorly.

Regarding locus‐specific filtering, Bowles et al. (2020) started with a typical and what we believe appropriate method of retaining loci that were genotyped in at least 80% of individuals within each population (i.e., −r 0.8 in the populations module of STACKs; Catchen, Hohenlohe, Bassham, Amores, & Cresko, 2013). However, they added an additional filtering component that resulted in the inclusion of many loci with 100% missing data in certain populations. Specifically, they retained loci if they met the 80% genotype rate criteria in 6/8 or 4/6 populations depending on which dataset they were analyzing (i.e., a locus could be missing 100% of data in 2 populations if there was <20% missing data in the other populations). This methodology resulted in a missing data pattern where the maximum percentage of missing data for a given locus was only ~40%, but certain populations contained large numbers of loci with 100% missing data (Figure S1). Most notably, over half of the loci were missing 100% of data in the PER03 population, and ~10% of loci were missing 100% of data in the CHA03 and TAK15 populations.

**FIGURE 3:**
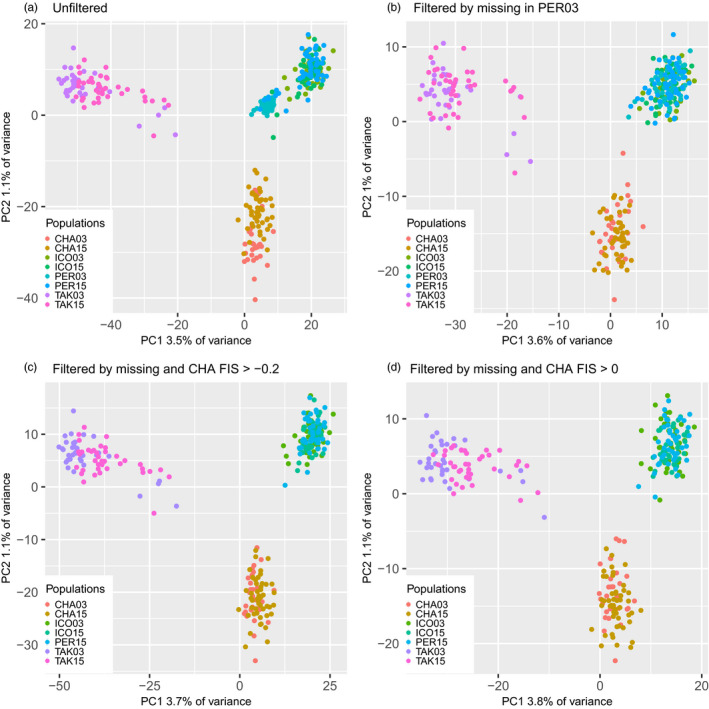
Individual‐based PCAs for all populations with four different datasets. Datasets are (a) unfiltered data downloaded from DRYAD, (b) dataset including only loci with >50% genotyping rate in PER03, (c) dataset including only individuals genotyped at >50% of loci and loci with *F*_IS_ > −0.2 in the CHA samples, and (d) dataset including only individuals genotyped at >50% of loci and loci with *F*_IS_ > 0 in the CHA samples. Each dot represents an individual, and individuals are color‐coded by population. See text for population abbreviations

To test the influence of these patterns of missing data on population structure, we conducted individual‐based PCAs in adegenet (Jombart, 2008) with the unfiltered dataset (i.e., the dataset presented in Bowles et al., 2020) and a dataset with only the loci that were genotyped in >50% of individuals in the PER03 population (Figure 3.). In the unfiltered dataset, the PER03 population is clearly separated from the PER15/ICO03/ICO15 cluster of points (Figure 3a). However, when loci missing >50% of data are removed, the PER03 individuals overlap completely with the PER15/ICO03/ICO15 cloud (Figure 3b). These results illustrate that the claim that the PER population was historically differentiated from ICO and genetically homogenized with the ICO population between 2003 and 2015 is false. PER03 is highly similar to ICO03/ICO15/PER15 when data are filtered properly, and the apparent structure between PER03 and these other three populations was caused by missing data. In fact, analysis of the same samples with microsatellites (Dupont et al., 2007) also supports our conclusion that PER03 and ICO03 are genetically homogenous, providing further evidence that structure between PER03 and ICO03 is a bioinformatic artifact caused by missing data.

**FIGURE 4:**
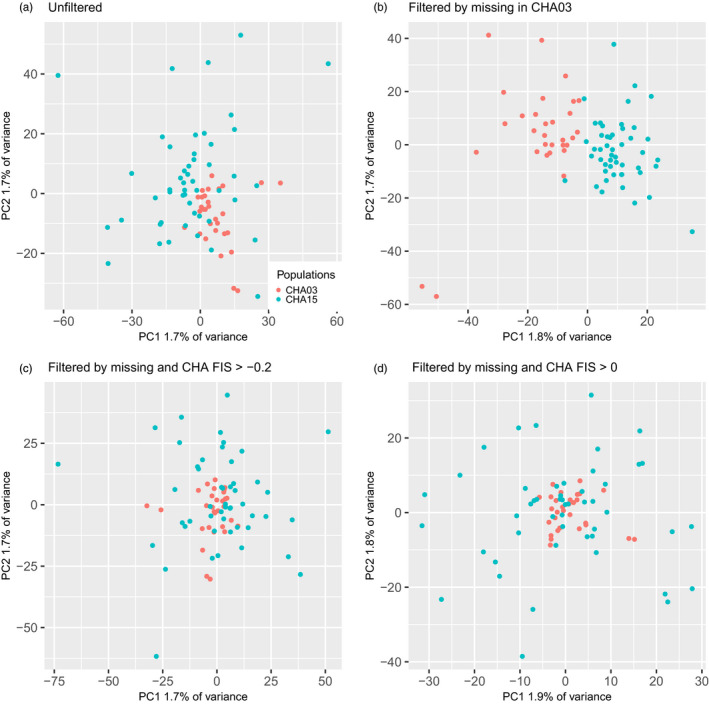
Individual‐based PCAs for the CHA populations with four different datasets. Datasets are (a) unfiltered data downloaded from DRYAD, (b) dataset including only loci with >50% genotyping rate in CHA03, (c) dataset including only individuals genotyped at >50% of loci and loci with *F*_IS_ > −0.2 in the CHA samples, and (d) dataset including only individuals genotyped at >50% of loci and loci with *F*_IS_ > 0 in the CHA samples. Each dot represents an individual, and individuals are color‐coded by population. See text for population abbreviations

**FIGURE 5:**
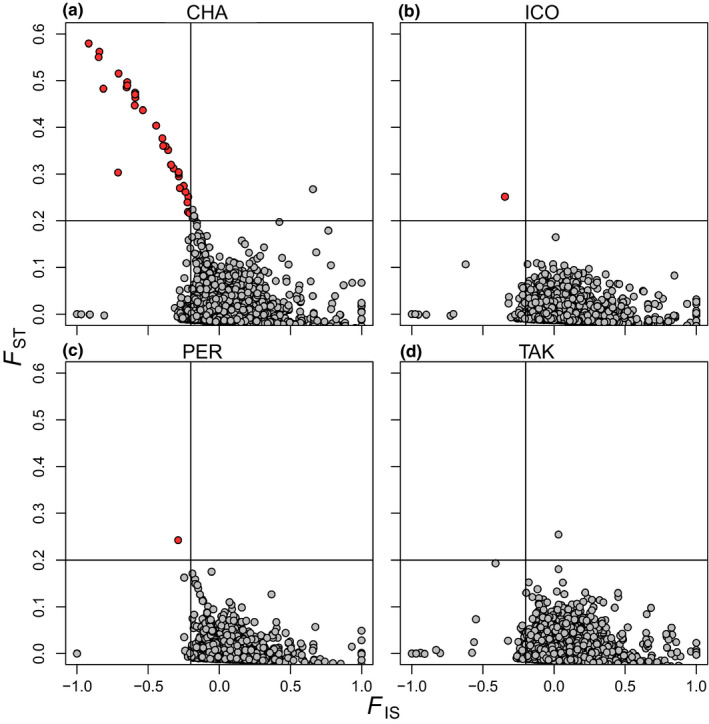
Distribution of *F*_IS_ and *F*_ST_ values for each population pair. *F*_IS_ values were calculated by pooling each sample from a given population pair. Loci with *F*_IS_ < −0.2 and *F*_ST_ > 0.2 (an arbitrary cutoff) are highlighted in red. Population abbreviations are found in the text

**FIGURE 6:**
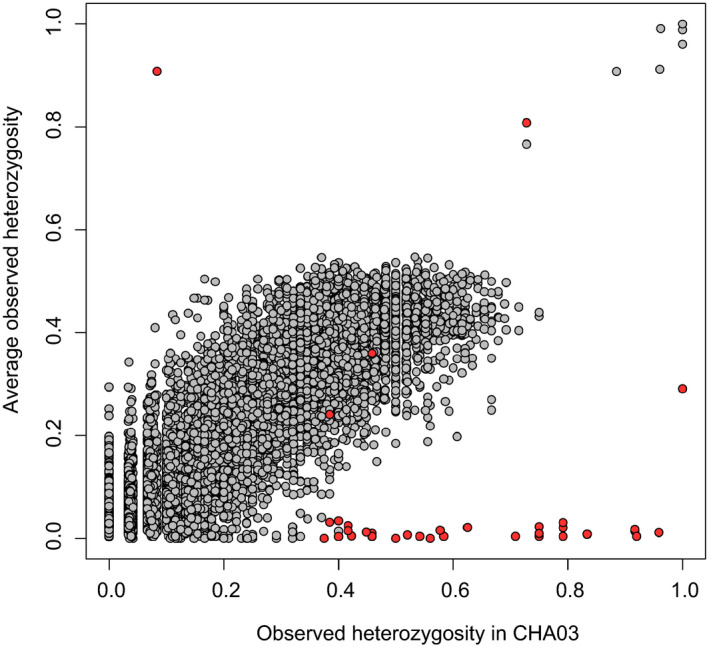
Observed heterozygosity in CHA03 (x‐axis) and all other populations (y‐axis). Loci with *F*_IS_ < −0.2 and *F*_ST_ > 0.2 in CHA samples are denoted in red

Missing data did not explain the apparent genetic differences between the CHA03 and CHA15 samples, as a similar PCA analysis with loci missing >50% in CHA03 removed still revealed strong structure between the two (Figure 4b). Rather, we believe population structure observed between the CHA03 and CHA15 samples was caused by inadequate bioinformatic filtering resulting in the retention of oversplit or undersplit loci. Adequate filtering is especially important when detecting loci under selection, as bioinformatic artifacts can often manifest as outlier loci with high *F*_ST_ values (O'Leary et al., 2018). The first step in vetting outlier loci used in our laboratory is visually examining their *F*_IS_ and *F*_ST_ values, as *F*_IS_ values that deviate substantially from zero can reveal either laboratory or bioinformatic errors (e.g., over or undersplitting) or interesting biological processes that create departures from Hardy–Weinberg equilibrium. When we were first exploring the Bowles et al. (2020) dataset, we sorted loci by *F*_ST_ and realized that many of the loci with the highest *F*_ST_s had highly negative *F*_IS_ values. We then isolated comparisons to population pairs sampled 12 years apart and graphed *F*_ST_ and *F*_IS_ values (WC 1984) calculated in Genepop V4 (Rousset, 2008; Figure 5). This analysis revealed that a large number of the high *F*_ST_ loci in the CHA comparison also displayed very negative *F*_IS_ values (Figure 5b), sharply contrasting results from other sample pairs (Figure 5). Notably, the CHA population pair has 70 loci with FST > 0.2 and FIS < −0.2, whereas all other population pairs have only 1 or 0 loci that fall into this category. These highly negative *F*_IS_ values are the result of extremely high locus‐specific heterozygosities in the CHA03 population (>90% in some cases) and extremely low locus‐specific heterozygosities in all other populations (<5% in some cases; Figure 6). In other words, some of these loci are nearly fixed heterozygotes in the CHA03 population while being nearly invariant in the other populations in the study including in the same population 12 years (i.e., <3 generations) later (CHA15). Put simply, this result is not biologically possible and is most likely a bioinformatic artifact.

Over and undersplit loci are difficult to detect and remove, as noted by O'Leary et al. (2018). However, O'Leary et al. (2018) recommend plotting heterozygosity and allele depth as a potential way to identify splitting errors. These metrics are efficiently visualized in HDplot (McKinney, Waples, Seeb, & Seeb, 2017), which is integrated into our typical filtering workflow. HDplot analysis revealed that about half of the high *F*_ST_–low *F*_IS_ loci that we identified deviated from typical patterns (Figures S2, S3). Most notably, a large number of the loci had allele ratios near 0.75, which could indicate over merging of loci. However, many of these loci fell within normal ranges and would have been retained by our typical filters of deviation between −5 and 5 (Figures S2, S3). These results support the conclusion by O'Leary et al. (2018) that over and undersplit loci are difficult to detect and remove. We believe that the most effective way to address this issue would be to re‐analyze the data in STACKs; however, the raw data for this study are not available. Therefore, we attempted to filter the data in different ways to determine the impact of these high *F*_ST_ low *F*_IS_ loci on patterns of population structure.

We analyzed four datasets to investigate the influence of filtering on genetic differentiation with an emphasis on the CHA population pair. These datasets were (1) the unfiltered data downloaded from DRYAD (8,728 loci), (2) a dataset with all SNPs retained but with individuals missing >50% of data removed (this removes all individuals from the PER03 population and up to 7 individuals from other populations), (3) a dataset with individuals missing >50% of data removed and with SNPs with *F*_IS_ < −0.2 in the CHA samples removed (8,560 loci), and (4) a dataset with individuals missing >50% of data removed and with SNPs with *F*_IS_ < 0 in the CHA samples removed (3,900 loci). First, we conducted individual‐based PCA as described above and found that the apparent genetic structure between CHA03 and CHA15 disappears when either *F*_IS_ filter is applied (Figures 3, 4). This is most observable in the PCAs including only CHA comparisons (Figure 4), where the strong structure observed between CHA03 and CHA15 in panels a and b disappears when loci are filtered by *F*_IS_ in both panels c and d.

We also evaluated genetic structure for these four datasets with estimates of pairwise *F*_ST_ (Weir & Cockerham, 1984) and exact tests of genetic differentiation in Genepop. *F*_ST_ estimates among most sample pairs were similar across datasets as expected (Table 1). However, pairwise *F*_ST_ values for the CHA03/CHA15 comparison decreased from 0.0036 for the unfiltered dataset to 0.0017 for the dataset excluding loci with *F*_IS_ < −0.2 to 0.0003 for the dataset excluding loci with *F*_IS_ < 0. Notably, when the data were filtered properly, the TAK reference population actually has a higher *F*_ST_ value between time points than any of the temporal replicates in the southern rivers. *F*_ST_ estimates were significant for the CHA03/CHA15 comparison with the unfiltered dataset and the dataset with low quality individuals removed, but were not significant with either of the datasets that were filtered based on *F*_IS_. In summary, filtering the dataset based on *F*_IS_ values produced consistent results for all sample pairs except for the CHA03/CHA15 pair, where genetic structure decreased substantially and was not significant when loci with low *F*_IS_ values were removed. These results demonstrate that the bias in this dataset extends below the artificial threshold of *F*_IS_ < −0.2 and illustrates the difficulty of filtering out bioinformatic artifacts such as over or undersplit loci.

**TABLE 1:** Pairwise *F*_ST_ estimates for four datasets

Pop	CHA03	CHA15	ICO03	ICO15	PER03	PER15	TAK03
Unfiltered							
CHA15	**0.0036**						
ICO03	**0.0218**	**0.0183**					
ICO15	**0.0214**	**0.0170**	**0.0001**				
PER03	**0.0210**	**0.0204**	**0.0020**	0.0012			
PER15	**0.0227**	**0.0186**	0.0006	−0.0004	**0.001**		
TAK03	**0.0568**	**0.0541**	**0.0777**	**0.0758**	**0.0803**	**0.0785**	
TAK15	**0.0623**	**0.0589**	**0.0834**	**0.0819**	**0.0838**	**0.0842**	0.0009
Missingness filter							
CHA15	**0.0036**						
ICO03	**0.0218**	**0.0183**					
ICO15	**0.0214**	**0.0168**	**0.0002**				
PER03	NA	NA	NA	NA			
PER15	**0.0225**	**0.0182**	0.0008	−0.0003	NA		
TAK03	**0.0568**	**0.0541**	**0.0775**	**0.0752**	NA	**0.0774**	
TAK15	**0.0623**	**0.0589**	**0.0832**	**0.0814**	NA	**0.0833**	0.0009
*F*_IS_ filter >−0.2							
CHA15	0.0017						
ICO03	**0.0204**	**0.0183**					
ICO15	**0.0197**	**0.0169**	0.0002				
PER03	NA	NA	NA	NA			
PER15	**0.0208**	**0.0182**	0.0008	−0.0003	NA		
TAK03	**0.056**	**0.0547**	**0.0782**	**0.076**	NA	**0.0781**	
TAK15	**0.0609**	**0.0594**	**0.0836**	**0.082**	NA	**0.0838**	0.0008
*F*_IS_ filter >0							
CHA15	0.0003						
ICO03	**0.0202**	**0.0183**					
ICO15	**0.0186**	**0.0161**	−0.0002				
PER03	NA	NA	NA	NA			
PER15	**0.0202**	**0.0183**	0.0008	−0.0003	NA		
TAK03	**0.0565**	**0.0532**	**0.0802**	**0.0778**	NA	**0.0796**	
TAK15	**0.0607**	**0.0572**	**0.0846**	**0.0832**	NA	**0.0844**	0.0013

Note

Values in bold are significant differentiated (*p* < .01). Datasets are unfiltered data downloaded from DRYAD, (b) dataset including only individuals genotyped at >50% of loci, (c) dataset including only individuals genotyped at >50% of loci and loci with *F*_IS_ > −0.2 in the CHA samples, and (d) dataset including only individuals genotyped at >50% of loci and loci with *F*_IS_ > 0 in the CHA samples.

Although the analyses described above illustrate that there is no significant genetic differentiation between temporally replicated sample pairs across all loci, it is still possible that some outlier loci differentiating the populations could exist. To investigate this hypothesis, we explored the distributions of *F*_ST_ values for each population pair with dataset four described above (Table 2, Figure S4). Distributions were quantified and visualized using all samples and with populations randomly subsampling to the minimum sample size in the study (*N* = 30). *F*_ST_ distributions for all three population pairs were highly similar (Figure S4). For example, when population sizes are normalized, the TAK (reference) and CHA (high exploitation) populations have similar numbers of SNPs with *F*_ST_s > 0.1, 0.15, and 0.2 (Figure S4). These results illustrate that there are no significant qualitative or quantitative differences in the *F*_ST_ distributions among the three population pairs.

**TABLE 2:** Characterization of *F*_ST_ distributions for CHA, ICO, and TAK population pairs

			*F*_ST_		*F*_ST_ range				
Population pair	N 03 pop	N 15 pop	Mean	Max	0–0.05	0.05–0.1	0.1–0.15	0.15–0.2	>0.2
CHA all samples	30	49	0.00003	0.27	2,967	116	21	3	1
ICO all samples	46	40	0.00006	0.16	3,628	69	4	1	0
TAK all samples	35	39	0.00100	0.18	3,008	109	14	2	0
CHA normalized	30	30	0.00030	0.21	2,840	142	35	5	2
ICO normalized	30	30	0.00006	0.18	3,486	138	23	4	0
TAK normalized	30	30	0.00100	0.23	2,946	118	29	4	1

Note

Data were generated with the dataset including only individuals genotyped at >50% of loci and loci with *F*_IS_ > 0 in the CHA samples. N is sample size, and populations in the “normalized” rows were randomly subsampled to 30, the minimum sample size in the dataset. Numbers in the columns beginning with *F*_ST_ are the number of loci in those categories.

In summary, we found that the inadequate filtering and bioinformatic abnormalities are likely responsible for the major genetic patterns reported by Bowles et al. (2020). Specifically, the temporal genetic homogenization between southern populations that they reported was caused by missing data and the genetic differentiation and outlier loci that they observed in the CHA population was likely caused by variable over or undersplitting of loci across populations. In fact, when data were filtered properly, the reference TAK population displayed the largest differentiation between time points rather than any of the high exploitation populations they examined. These results illustrate the extreme importance of thorough data filtering.

## 5 CONCLUSIONS

We re‐analyzed the age/growth and genomic data presented in Bowles et al. (2020) and conclude that the data do not support the authors conclusion of phenotypic and genetic changes caused by harvest. The age/growth data suggest that walleye populations in Lake Mistassini may have decreased slightly in size‐at‐age over the study period, but this was true for all populations, including the reference population with lower exploitation. This result suggests that potential declines in walleye size‐at‐age is likely due to environmental factors that are impacting walleye populations in this lake regardless of exploitation rates, and/or that exploitation was similar across populations during the study period, meaning the Takwa River was not a viable reference population. Additionally, we discovered the genetic homogenization and divergence documented by the authors was the result of bioinformatic artifacts caused by inadequate data filtering. Specifically, when data were filtered properly using well‐established methods, population structure between time points disappeared. Conclusively demonstrating that observed changes in fish populations are the results of harvest is difficult, as these changes can be caused or influenced by myriad biotic and abiotic factors. Our comment illustrates the potential pitfalls associated with studying harvest‐induced changes in fish populations and highlights the importance of conducting robust analyses and data filtering before concluding that populations are influenced by harvest.

## Supporting information

Fig S1

Fig S2

Fig S3

Fig S4

Table S1

Supp_file S1

Supp_file S2

Supp_file S3

Supp_file S4

## Data Availability

All scripts and input files used in this analysis are available as supplementary material or will be uploaded to DRYAD upon acceptance.
