# Supplementary figures and images for: Incomplete bioinformatic filtering and inadequate age and growth analysis lead to an incorrect inference of harvested‐induced changes
Source: Evol Appl. 2020 Sep 12;14(2):278–89. doi: 10.1111/eva.13122 (PMC7896720; doi:10.1111/eva.13122)

# all populations

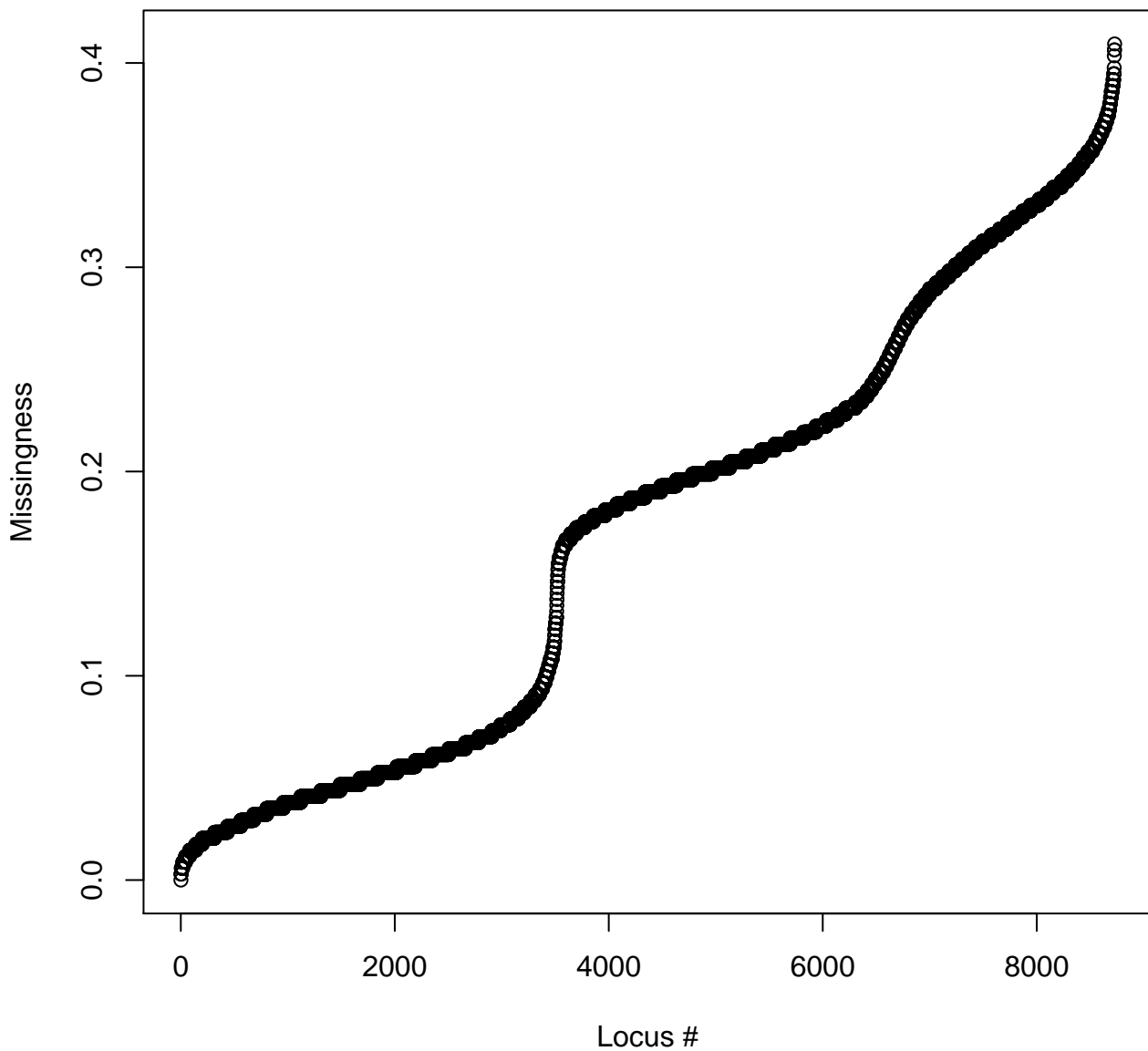

# CHA03

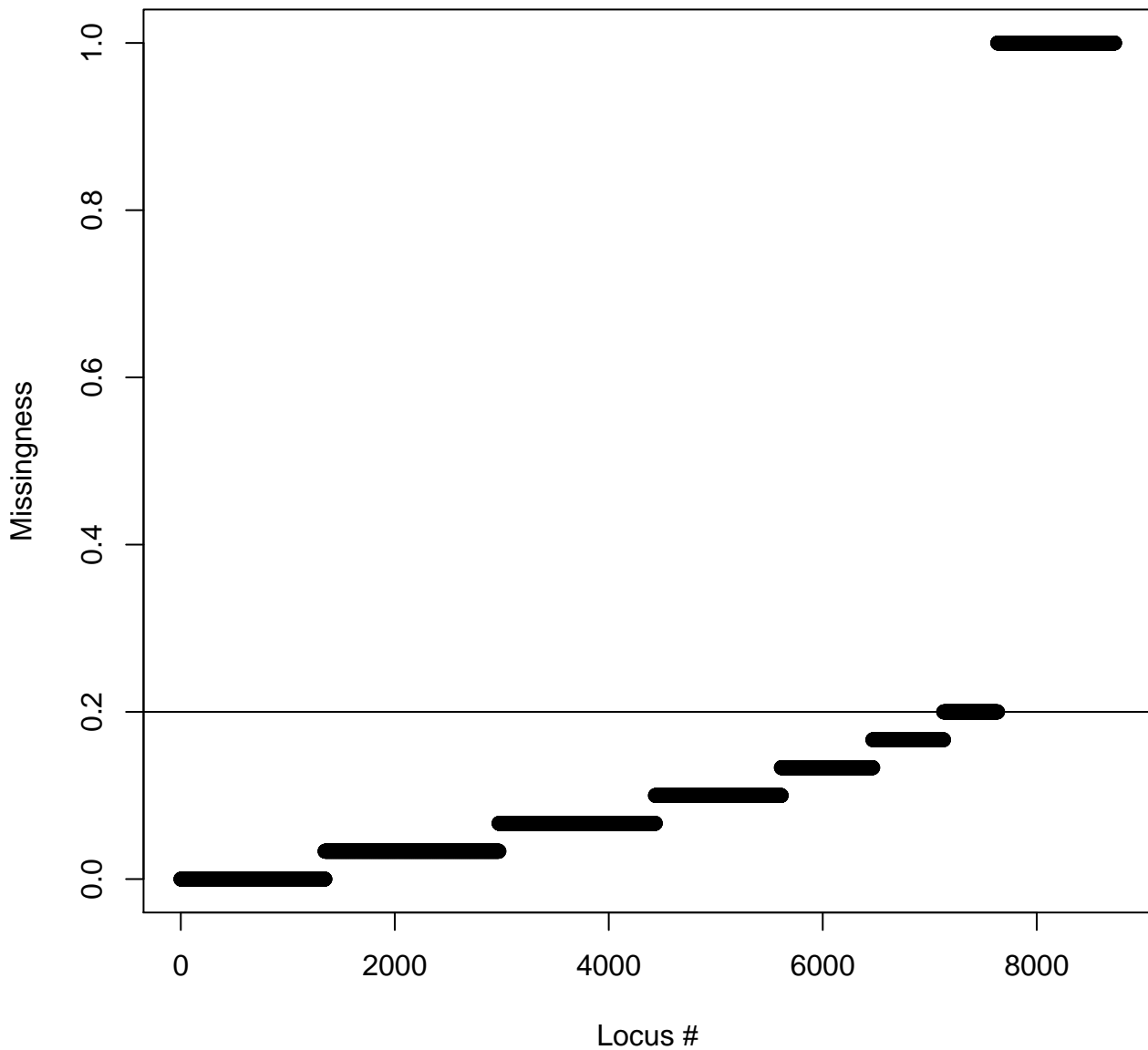

# CHA15

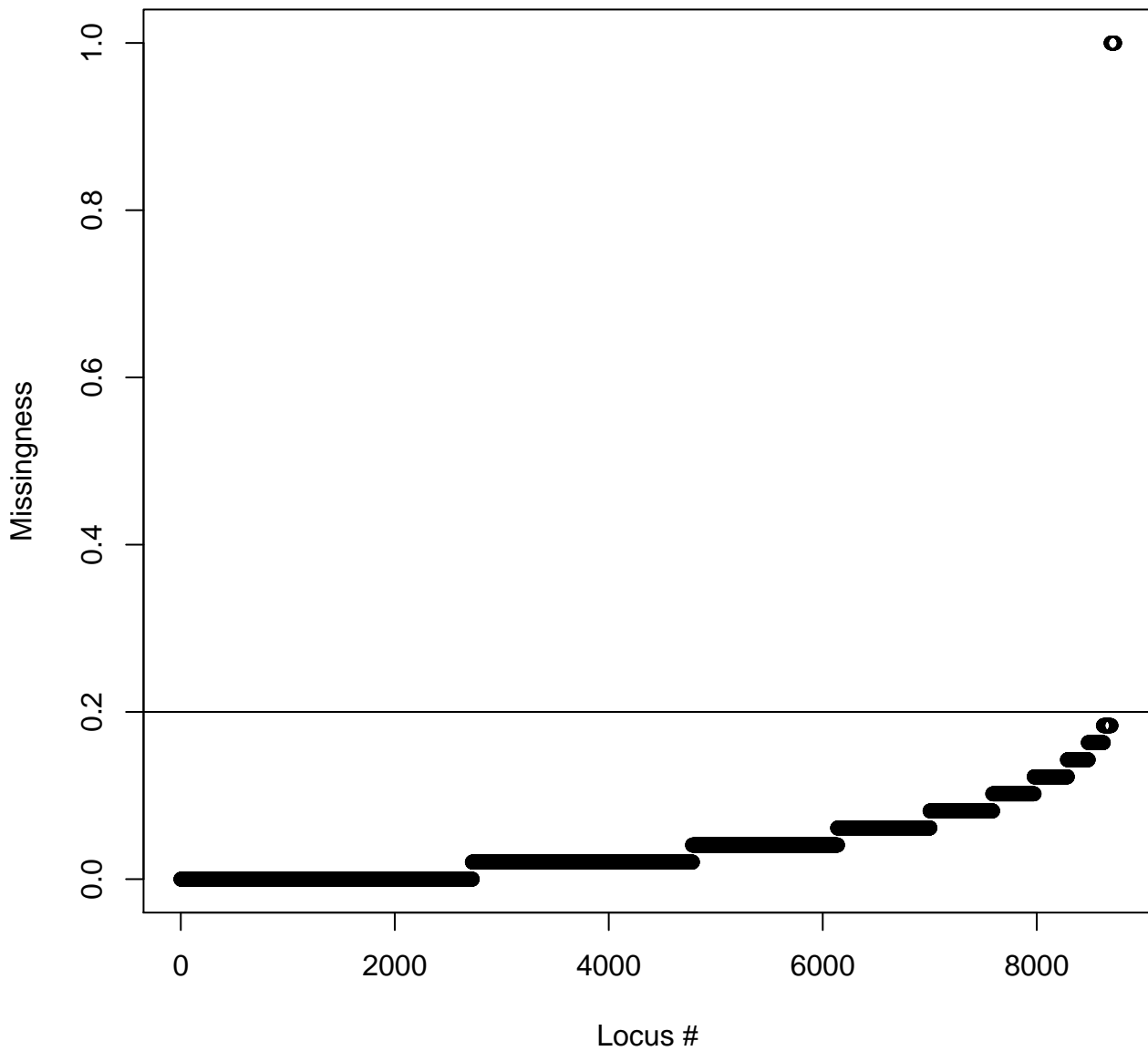

# ICO03

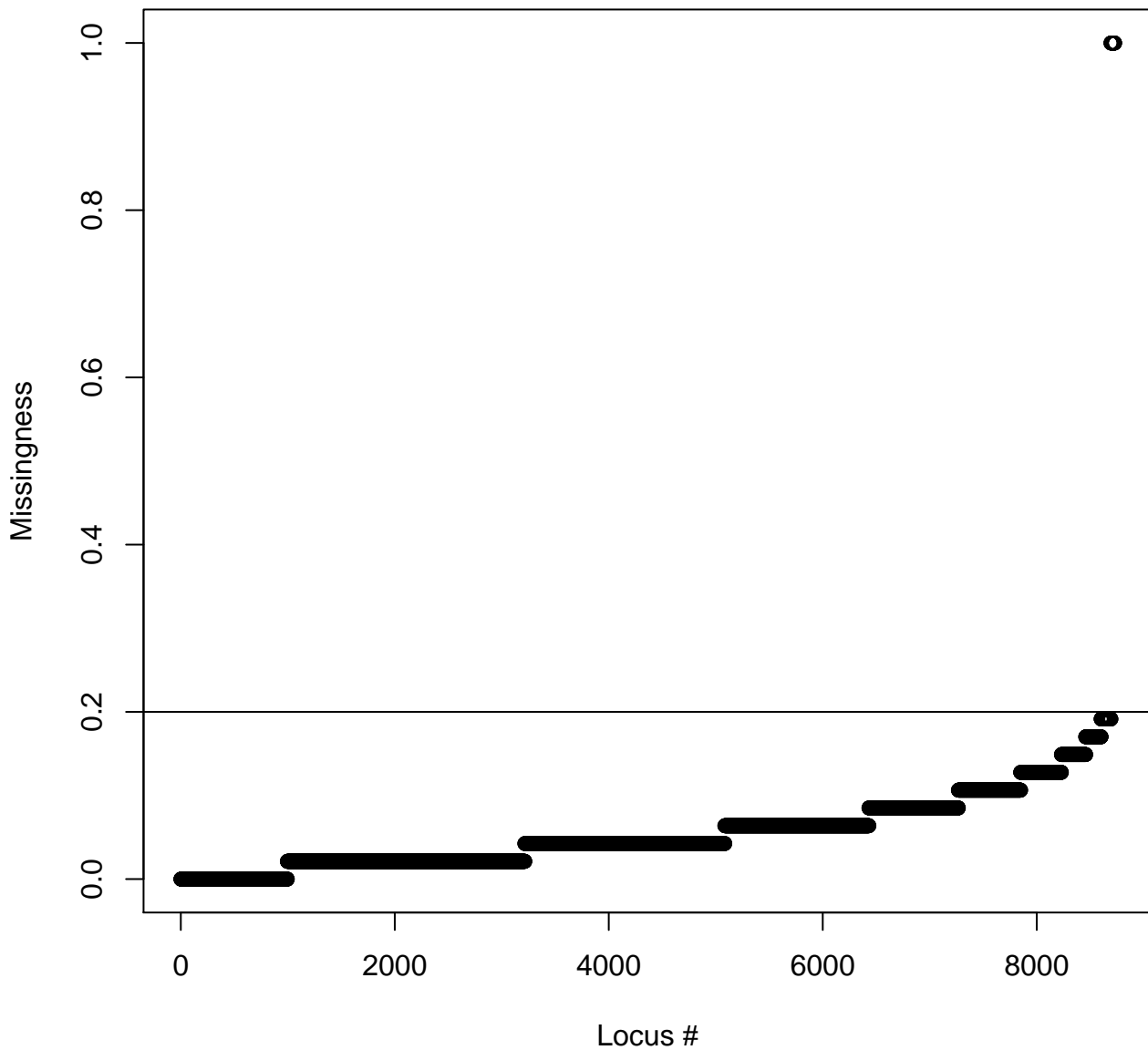

# ICO15

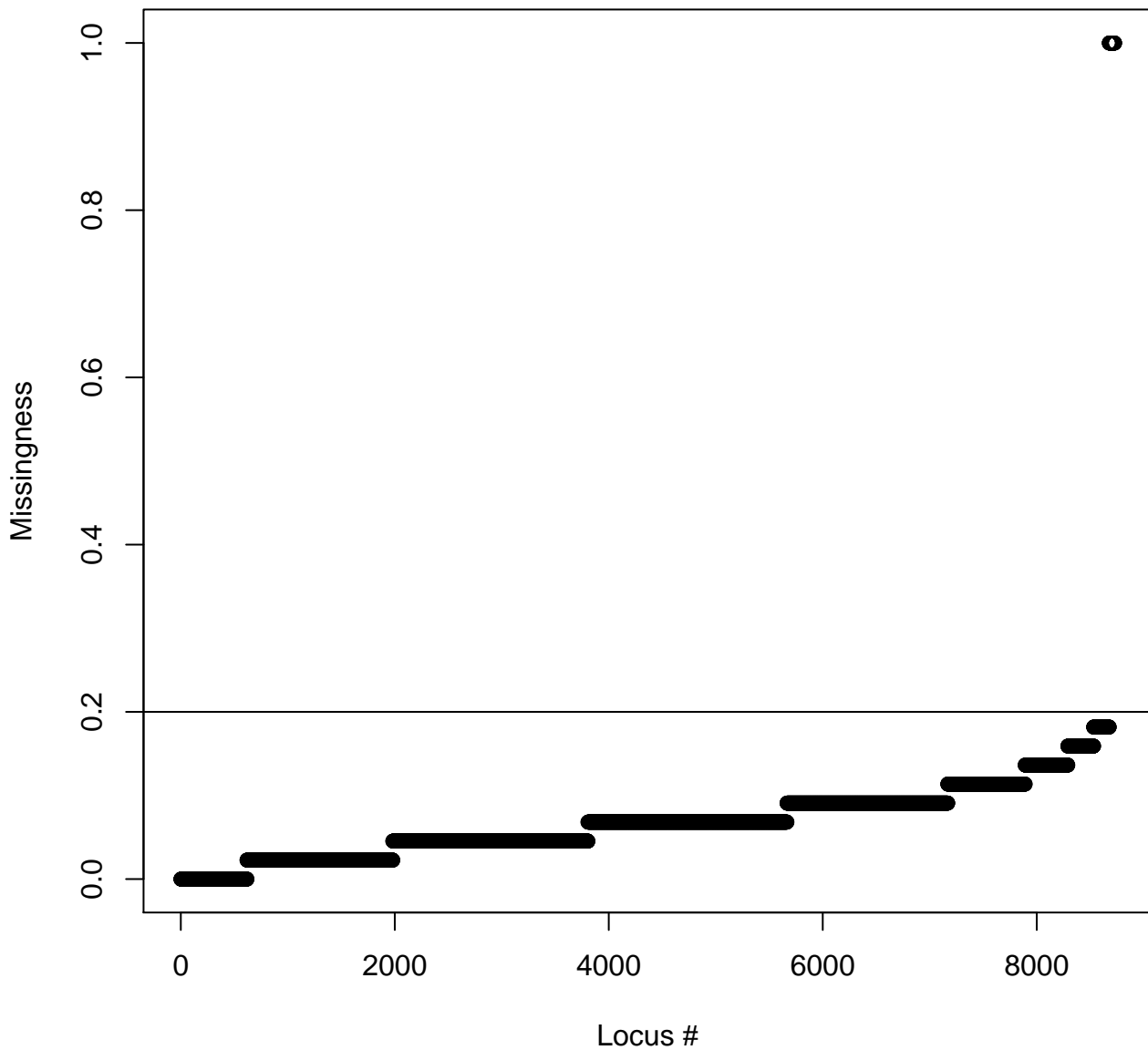

# PER03

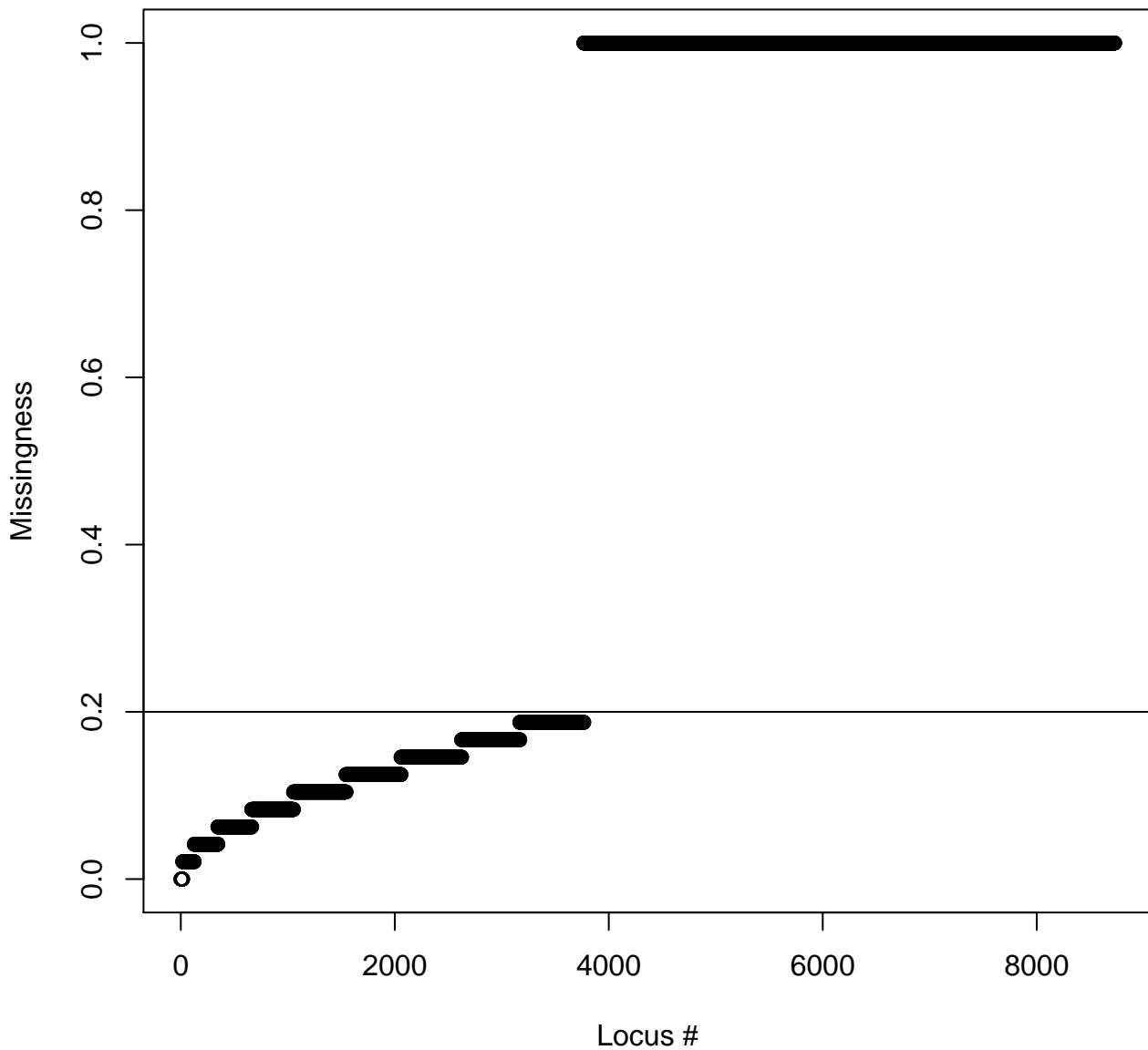

# PER15

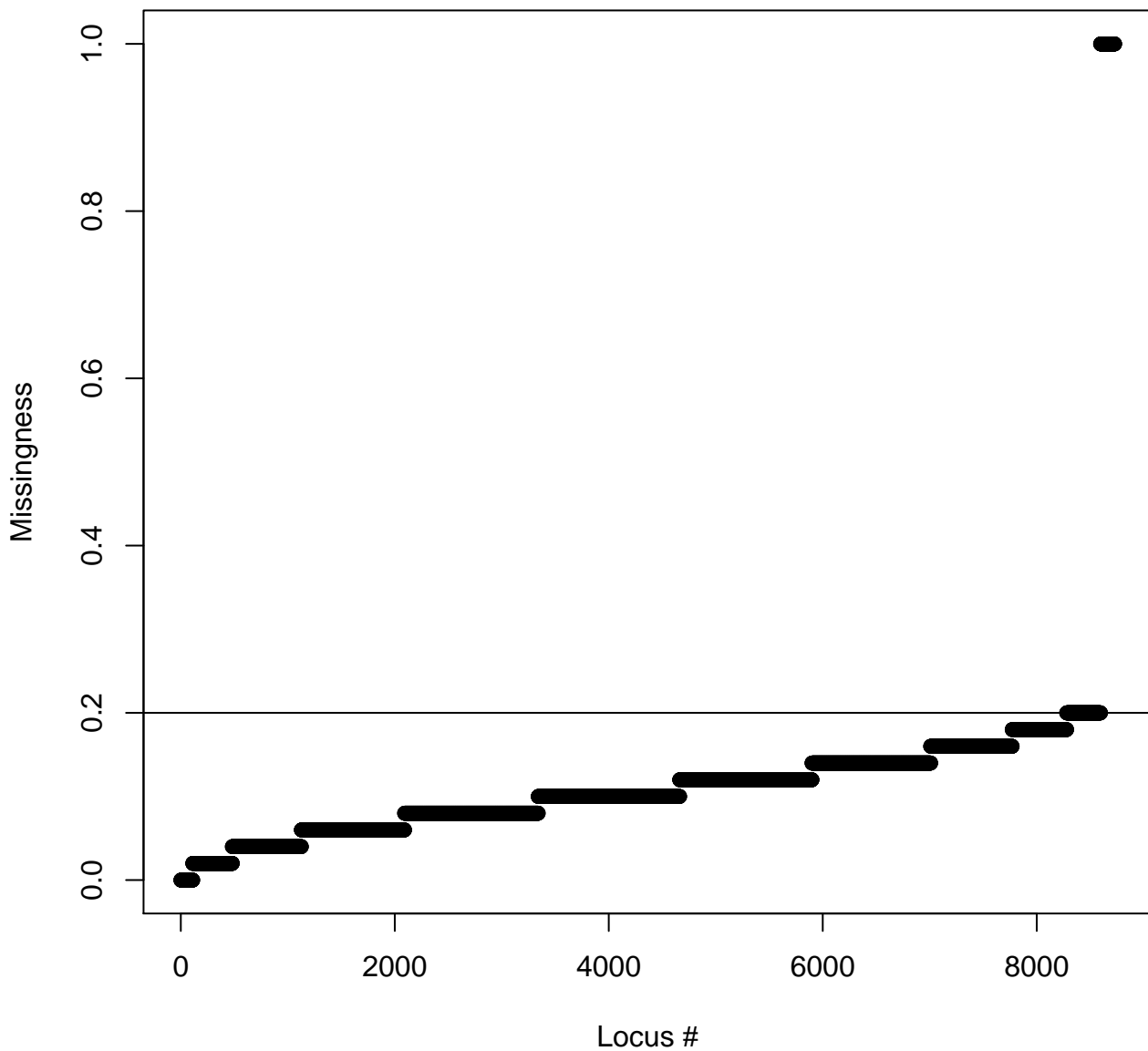

# TAK03

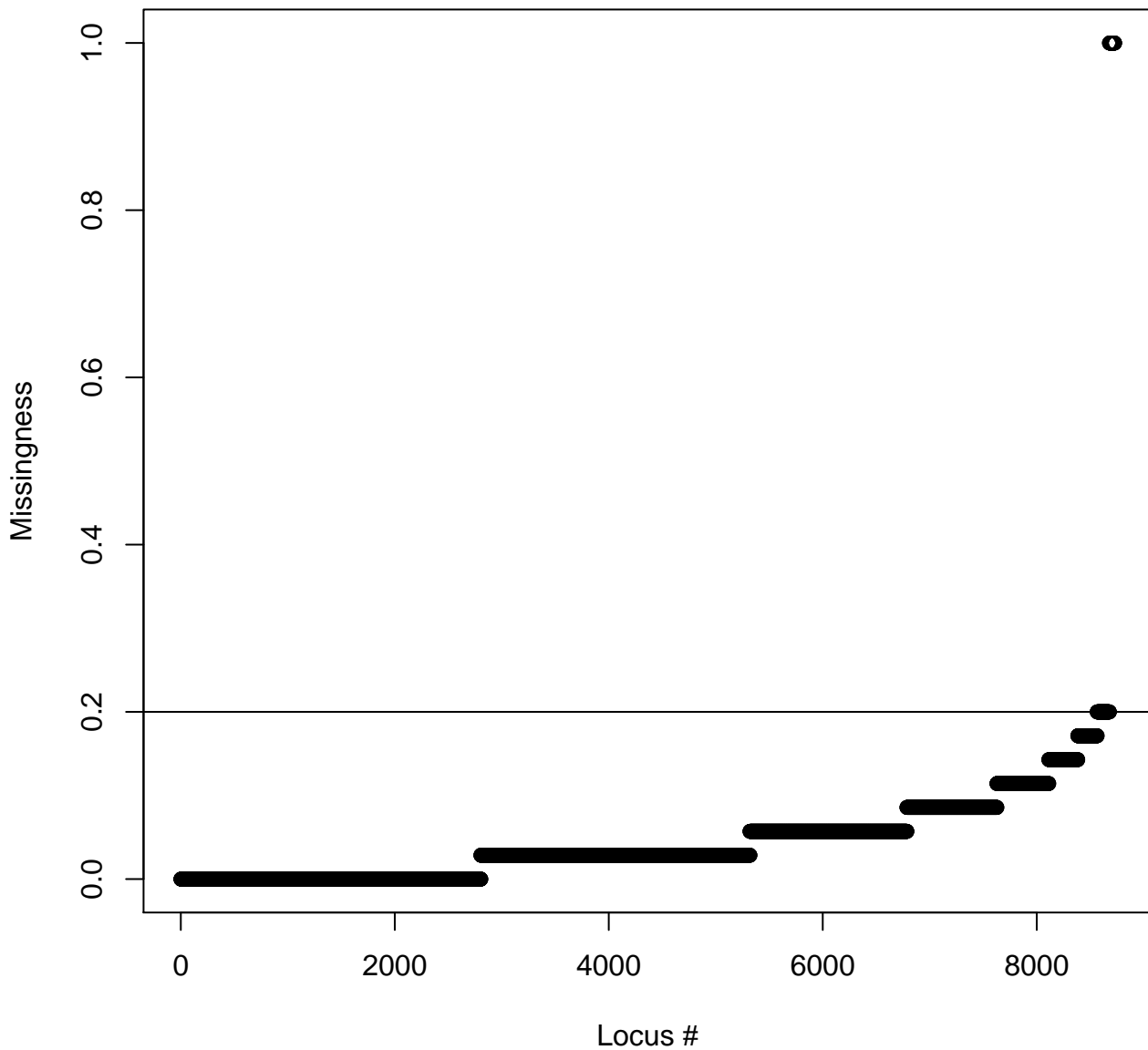

# TAK15

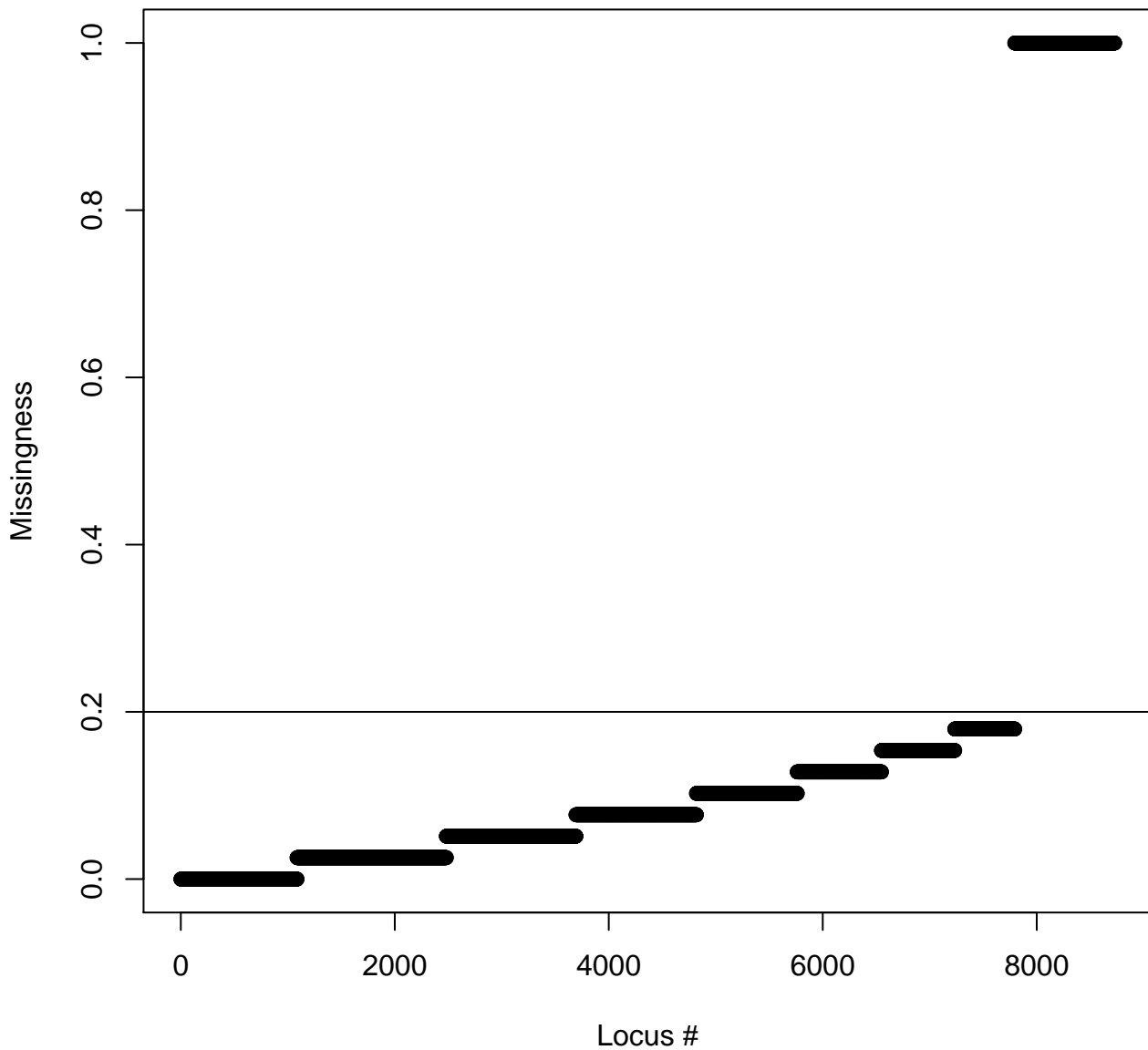

Supplement: Supplementary file 1 — Fig S1 [file EVA-14-278-s001.pdf]

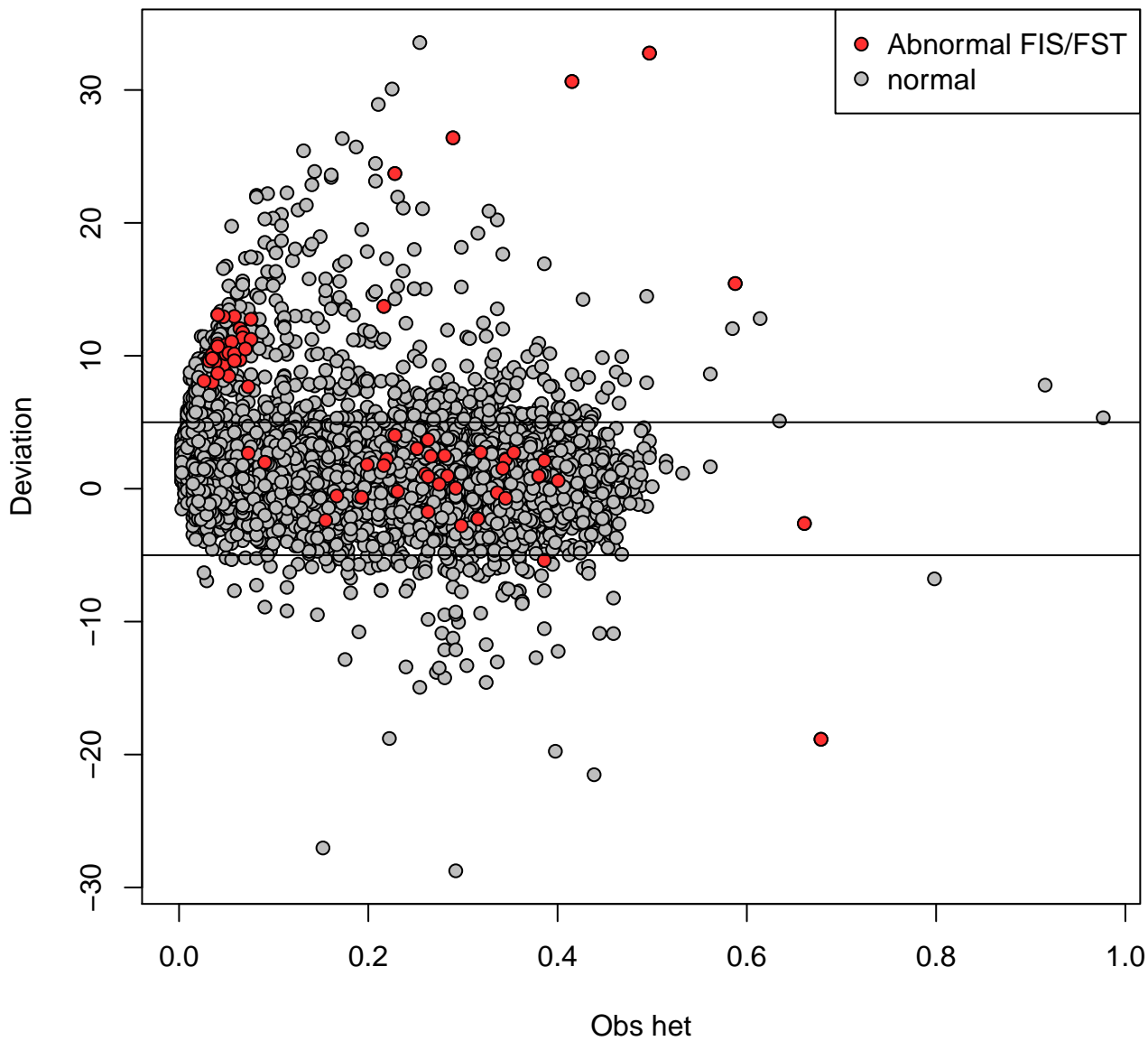

Supplement: Supplementary file 2 — Fig S2 [file EVA-14-278-s002.pdf]

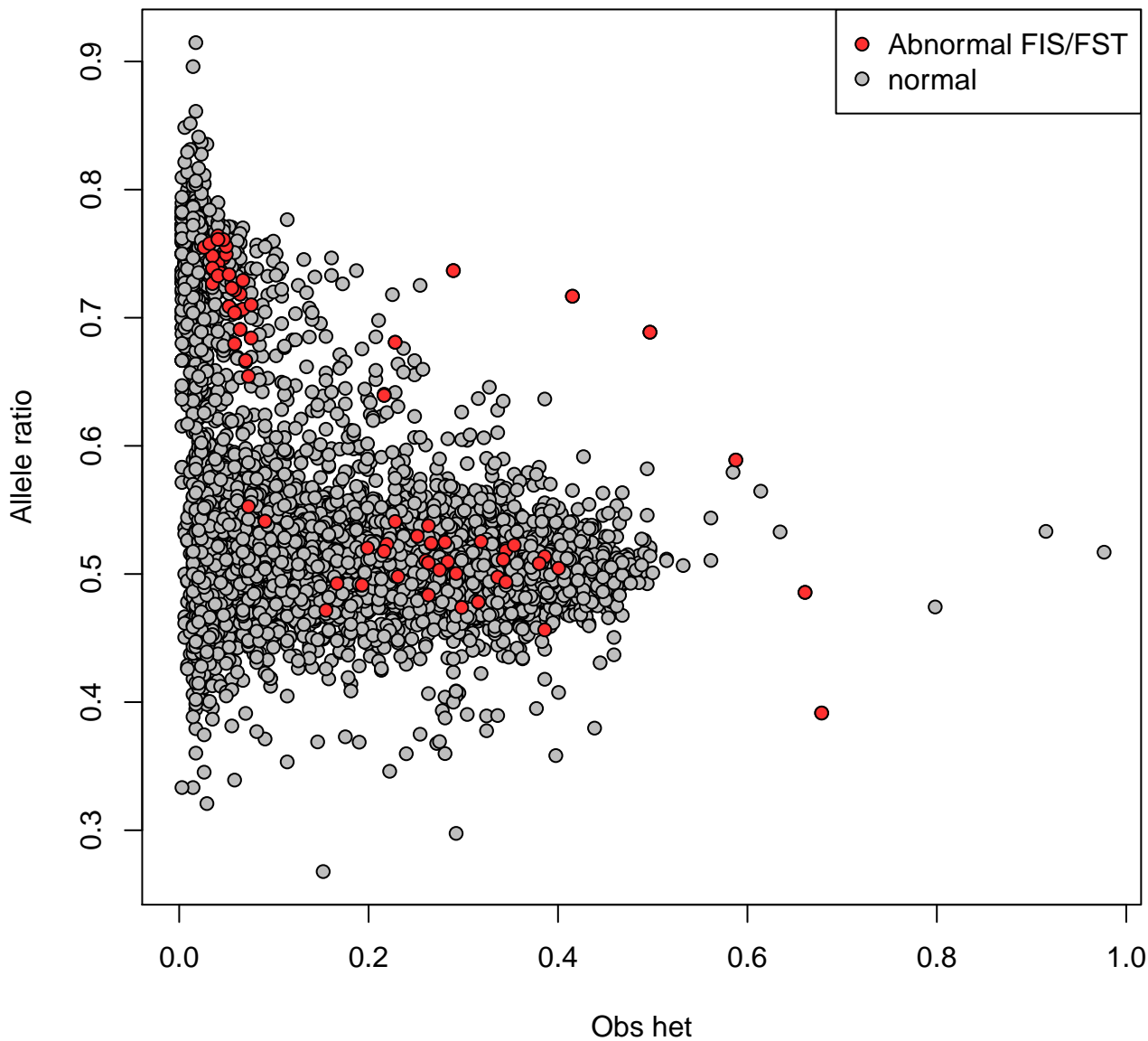

Supplement: Supplementary file 3 — Fig S3 [file EVA-14-278-s003.pdf]

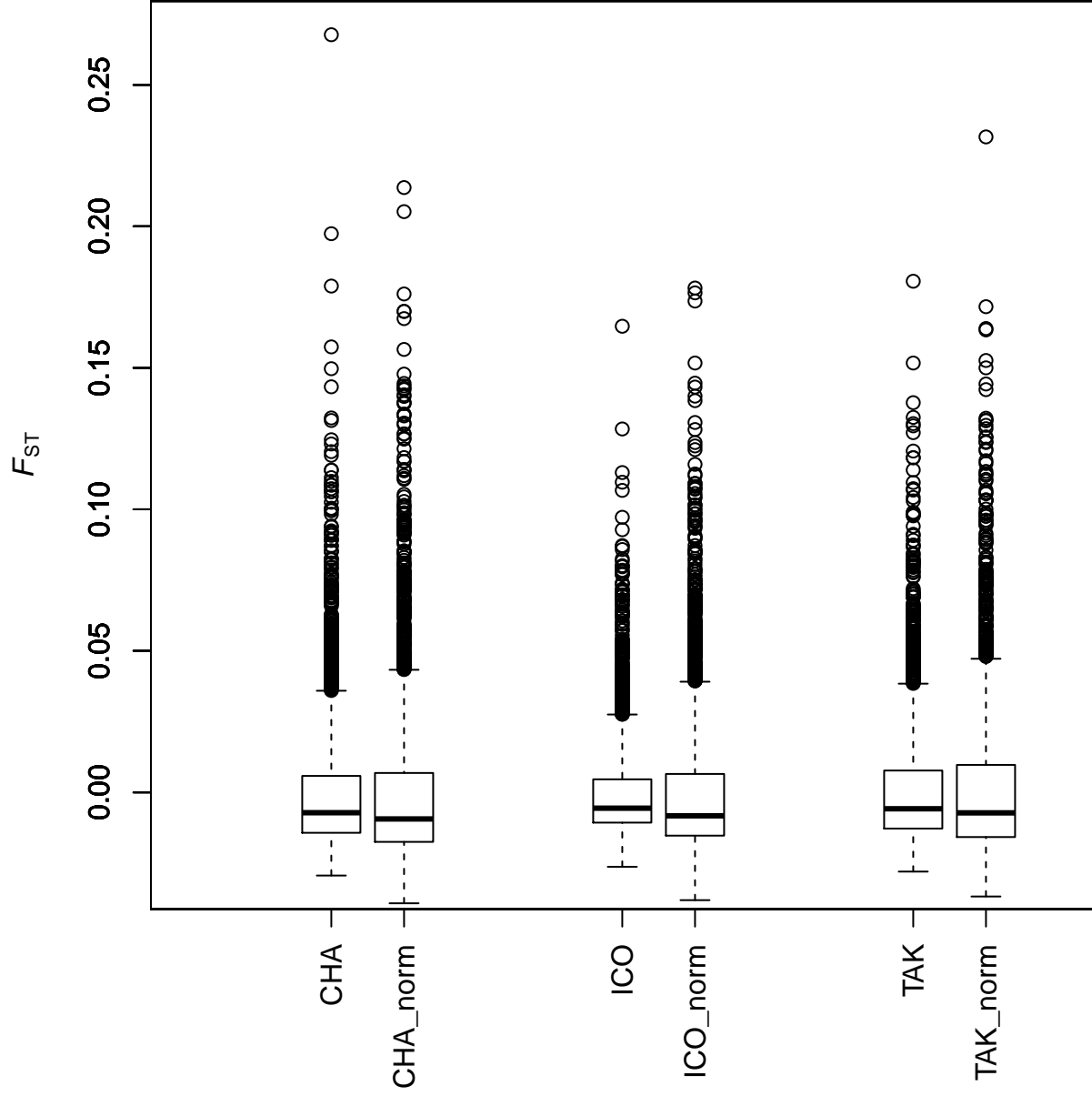

Supplement: Supplementary file 4 — Fig S4 [file EVA-14-278-s004.pdf]
